# Cognitive status predicts preoperative instruction compliance

**DOI:** 10.3389/fnagi.2023.1081213

**Published:** 2023-01-26

**Authors:** Yasuko Mano, Porus Mistry, Khoa Tran, Benjamin Wright, Cristin Malekyan, Tatyana Gurvich, Carolyn Kaloostian, Arash Motamed, Justyne Decker

**Affiliations:** ^1^Department of Anesthesiology, Keck Medical Center, University of Southern California, Los Angeles, CA, United States; ^2^Department of Family Medicine, Keck Medical Center, University of Southern California, Los Angeles, CA, United States; ^3^Department of Pharmacy, Keck Medical Center, University of Southern California, Los Angeles, CA, United States

**Keywords:** perioperative brain health, perioperative neurocognitive disorder, surgery and aging, quality improvement, geriatric surgical care

## Abstract

The most common postoperative complication for older adults is perioperative neurocognitive disorder (PNCD). Its greatest risk factor is preoperative cognitive impairment. Cognitive impairment also predicts higher likelihood of postoperative complications. While the cause of disparity in outcomes is likely multifactorial, the ability to correctly follow perioperative instructions may be one modifiable component. The purpose of this study was to determine whether cognitive impairment led to reduced preoperative instruction compliance and if so, identify barriers and enact a tailored care-plan to close the gap. Our preoperative clinic implemented routine Mini-Cog screening to identify older (age ≥ 65) surgical patients at increased risk. All patients received the same instructions and, on day of surgery, were surveyed to determine correct execution of *nil per os* guidelines, chlorhexidine wipe use and medication management. Data was stratified by cognitive status to evaluate whether impairment predicted instruction execution. Feedback from patients and families were compiled. Of those who screened negative for impairment, 68% correctly followed instructions, while 84.2% of those impaired struggled with ≥1 instruction(s); impaired patients were more likely to incorrectly follow instructions (OR = 10.5, *p*-value = 0.001). Areas for change were identified and team-based solutions were enacted with additional support for those with impairment. We found a clear difference in correct execution with respect to cognitive status. By improving instructions as an institution and adding additional support for those with impairment, the compliance gap was significantly reduced. Targeting perioperative instructions and tailoring care in this population may be one modifiable component in the outcome disparity they face.

## Introduction

Perioperative neurocognitive disorder (PNCD) encompasses delirium and delayed neurocognitive recovery—“brain fog,” or difficulty with concentration and memory that may occur postoperatively (ASA and AARP, 2018; Berger et al., 2018). It is the most common postoperative complication to affect older adults. Development of PNCD can last beyond the hospitalization, and even with resolution is not without consequence, as there is increased risk of perioperative complications and for developing dementia later in life (Witlox et al., 2010; Sprung et al., 2017). These experiences can be distressing for patients and their families and, in some cases, be life-altering.

The greatest risk factor for developing PNCD is the presence of preoperative cognitive impairment. In addition to the increased risk of cognitive complications, patients with impairment are at increased likelihood of other postoperative complications, increased length of stay, and being discharged to a place other than home (Culley et al., 2017). For this reason, the American Geriatrics Society, American College of Surgeons (Chow et al., 2012; Mohanty et al., 2016), and American Society of Anesthesiologists Perioperative Brain Health Initiative (n.d.) recommend routine preoperative cognitive screening to identify those at increased risk of developing PNCD and propose tailored perioperative management for the older, at-risk adult.

The cause of this outcome disparity is likely multifactorial consisting of both fixed and modifiable components. There are key action items that surgeons, anesthesiologists and the healthcare system can take to tailor the surgical care for the older at-risk patient, such as preoperative cognitive screening, reducing high risk medication administration, ensuring adequate pain control, prompt returning of cognitive aids—hearing aids, glasses, dentures, promoting sleep hygiene and protecting sleep, postoperative delirium assessments, and emphasizing non-pharmacologic interventions when delirium occurs (Peden et al., 2021). These actions aim to mitigate risk and help create an age-friendly perioperative care pathway. Nevertheless, how well do our patients with cognitive impairment navigate our care plan and execute the instructions or recommendations we make? The patient’s ability to interpret and correctly follow perioperative instructions could be another modifiable component. The aim of this study was to determine whether the presence of cognitive impairment or frailty led to reduced preoperative instruction compliance and, if so, determine whether it is modifiable through identification of barriers to correct execution and through enacting of a tailored care plan to close the gap.

## Methods

In line with perioperative societal recommendations (Mohanty et al., 2016; ASA and AARP, 2018), the preoperative clinic at Keck Medical Center of the University of Southern California implemented routine cognitive and frailty screening to identify older surgical patients who were at increased risk of PNCD. The Mini-Cog and FRAIL scale were chosen as screening tools for this study based on the fact that they are easy to administer and could be completed in parallel with other clinic duties. A documented Mini-Cog score of ≤2/5 was considered positive for preoperative cognitive impairment, and > 5 points on the FRAIL scale was considered positive for frailty. All patients were given the same preoperative instructions regarding *nil per os* (NPO) – when to stop eating and drinking prior to surgery, the usage of chlorhexidine wipes, and which medications to continue or hold before surgery.

**Figure 1:**
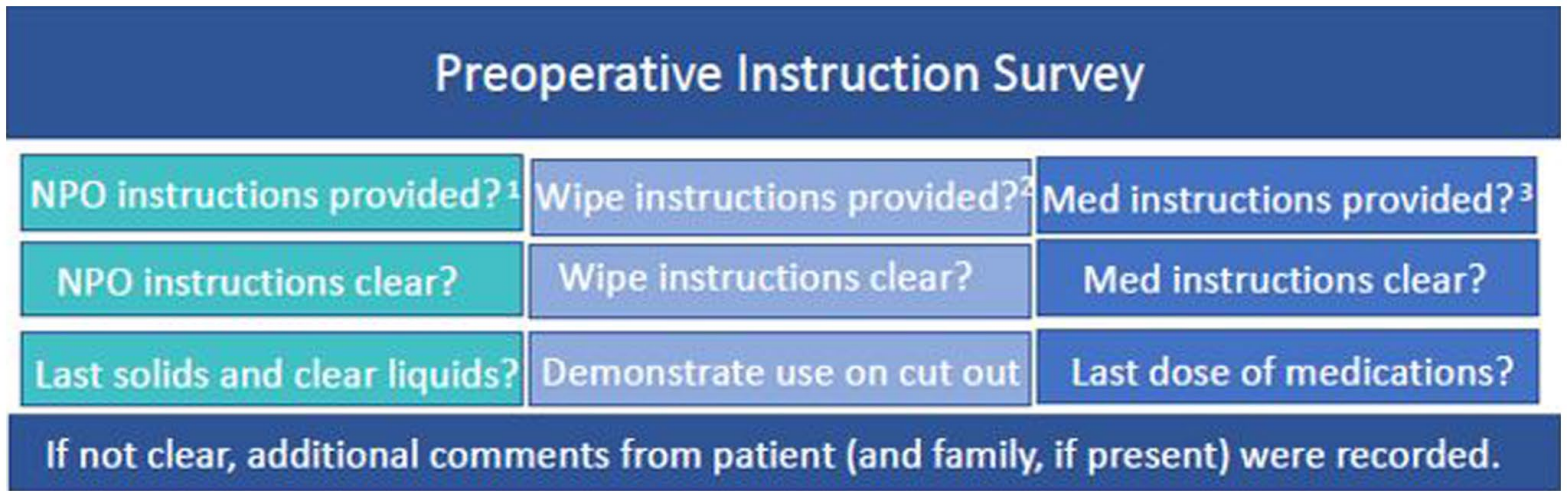
Preoperative Instruction Survey Questions. ^1^NPO instructions: no solid food within 8 h of scheduled surgical time, drink 16oz. of water at least 2 h prior to scheduled surgery time. ^2^CHG wipe instructions: handout was reviewed in clinic and provided to take home. ^3^Medication instructions included in this study: antihypertensives, blood thinners and diabetic medications.

On the day of surgery, all patients age ≥ 65 were met in the preoperative holding area and asked to participate in a 9-question survey (Figure 1) about the preoperative instructions they received. Patients and family, if present, could additionally provide further comment if they felt that the preoperative instructions were not easy to understand or unclear. Compliance with preoperative instructions was determined based on correctly following NPO directions—drinking 16oz of water at least 2 h prior to scheduled surgery time and no solid food within 8 h of scheduled surgical time, demonstration of correct chlorhexidine wipe usage, and correct medication management. To assess correct medication management by the patient, the last dose of commonly prescribed and frequently altered medications—antihypertensives, blood thinners and diabetic medications—was recorded and compared to the instructions that were provided. Patients were excluded from the study if they did not go to the preoperative clinic prior to surgery, were undergoing emergent procedures, or declined to participate.

The collected data was stratified by preoperative cognitive and frailty status to evaluate whether there was a correlation between preoperative status and correct execution of instructions. Feedback from patients and their families were compiled to identify compliance barriers, and targeted changes were made to the delivery of our preoperative instructions accordingly.

## Results

Of the 97 patients initially surveyed, 28 patients met exclusion criteria and were not included in the study. Of the remaining 69 patients in the study, 50 patients screened negative for the presence of preoperative impairment and frailty, and 68% of these patients correctly followed all preoperative instructions. Of the 19 patients who screened positive for preoperative cognitive impairment or frailty, 84.2% struggled with one or more of the preoperative instructions, leading to inadequate NPO status, incorrect usage of chlorhexidine wipes, and/or inappropriate preoperative medication management (Table 1). Patients with cognitive impairment or frailty were more likely to have difficulty correctly following preoperative instructions (OR = 10.5, *p*-value = 0.001). While recognizing the limitation of our small sample size, we strongly felt that the number of patients who struggled with preoperative instructions both without, and especially with, cognitive impairment did not warrant further data collection and instead required action—identifying targets for improvement in the preoperative instructions and trialing solutions to address them.

**Table 1:** Preoperative instruction compliance among patients with and without cognitive impairment or frailty (baseline).

	No impairment	Impairment	*p*-Value (OR)
Age	73.54	75.79	0.903
ASA physical status	2.66	2.89	0.14
Correct	34	3	0.001 (10.5)
Missed 1 or more	16 (32.0%)	16 (84.2%)	

**Figure 2:**
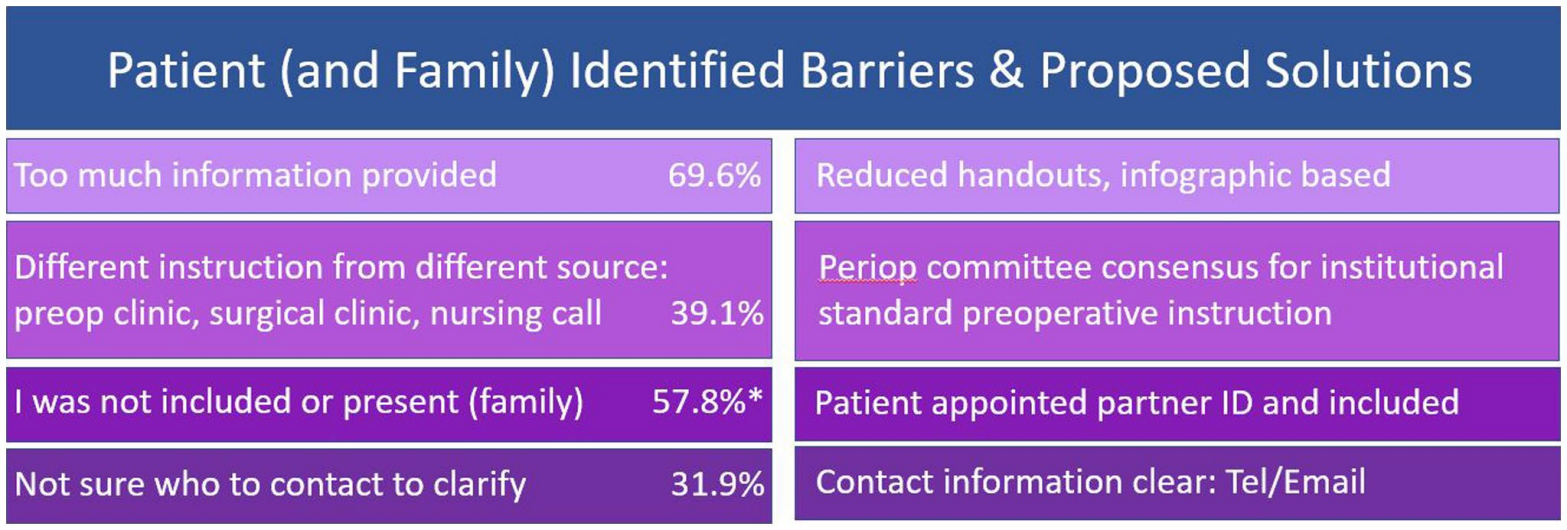
Identified barriers to correct execution of preoperative instructions with corresponding proposed team-based solutions. The frequency of each identified barrier among all patients is included. The frequency of reporting inclusion or presence of a family member as a barrier was only included for those with preoperative cognitive impairment.*

For each of the barriers to instruction compliance that were identified through the preoperative surveys, a team-based solution was proposed and enacted (Figure 2). As the feedback revealed that the preoperative information provided to patients was too abundant, the number of handouts were reduced from 15 to 3 and their format was changed to become more infographic-based. Given the confusion caused by the lack of consistency in instructions patients received from different healthcare personnel involved in their perioperative care—the preoperative clinic, surgical clinic, and same day surgery nursing call—the issue was brought up to the perioperative committee, who then reached consensus for institutional standardization of preoperative instructions to prevent differing instructions. Some patients were unaware of whom to contact to ask clarifying questions regarding preoperative instructions, so specific contact information (telephone, email) was provided for the surgical coordinator and preoperative clinic nurse. Finally, for those with cognitive impairment or frailty, a patient-appointed family member or friend was identified as their surgical partner and included in all instructions and recommendations.

After implementation of the aforementioned changes, 387 new patients were surveyed; 87 of these patients met exclusion criteria and were not included in the study. Of the remaining 300 patients in the study, 225 patients screened negative for the presence of preoperative impairment or frailty, and 88.0% of these patients correctly followed all preoperative instructions. Notably, of the 75 patients who screened positive for preoperative cognitive impairment or frailty, 78.7% were now able to correctly follow all preoperative instructions with assistance from their surgical partner (Table 2). Patients with cognitive impairment or frailty were still more likely to have difficulty correctly following preoperative instructions; however, that gap had significantly narrowed (OR = 1.99, *p*-value = 0.046).

**Table 2:** Preoperative instruction compliance among patients with and without cognitive impairment or frailty (post-intervention).

	No Impairment	Impairment	*p*-Value (OR)
Age	74.33	73.82	0.56
ASA physical status	2.76	2.88	0.06
Correct	198	59	
Missed 1 or more	27 (12.0%)	16 (21.3%)	0.046 (1.99)

## Discussion

Despite the small sample size used for this pilot study, we found a clear difference in correct execution of preoperative instructions among our older surgical patients with respect to cognitive and frailty status. Those who screened positive for cognitive impairment or frailty were more likely to have difficulty following preoperative instructions.

This study also revealed that changes in the delivery of preoperative instructions needed to be made at the institutional level. Areas for change were identified through patient and family feedback. By addressing these barriers, there was significant improvement in compliance with preoperative instructions among our overall patient population and, with the addition of a tailored instruction plan, we were able to significantly close the gap in the difference in execution among patients with cognitive impairment—the key being inclusion and an active role for the patient appointed partner through the preoperative process.

A limitation of this study was, as previously mentioned, the small sample size used. Particularly in the initial stage of the study, only 69 patients were included in the study. Although it would have been ideal to collect more data at this stage, there was clear evidence that patients were struggling to comply with instructions regardless of cognitive status, it was deemed necessary to intervene at this point and take action to address the barriers to compliance that were brought up in the surveys. Given the fact that the difference in preoperative instruction compliance between the two groups was statistically significant despite the small sample size, the conclusion regarding the association between preoperative cognitive status and instruction compliance could still be made.

Ultimately, the goal is to improve the surgical outcomes for all older adults and reduce the discrepancy in outcome for those with cognitive impairment. We recognize that optimizing preoperative instruction compliance is one small component of perioperative care and that this improvement alone is unlikely to lead to a significant change to the outcome disparity they face. However, this is one small step in the right direction. What if we were to go through the entire perioperative care plan identifying all of the areas that those with impairment are struggling with and enacted similar modifications to close the gap? These changes, in combination with enacting the aforementioned healthcare system key actions to provide age-friendly surgical care, may collectively lead to improved outcomes for the older at-risk adult. Future studies are directed toward identification of these additional perioperative barriers those with cognitive impairment face, tailoring their care accordingly, and assessing for improvement in bridging the gap.

Perioperative neurocognitive disorder is the most common postoperative complication for older adults. Preoperative cognitive impairment increases the risk for development of PNCD as well as other postoperative complications. The cause of this disparity in outcome for those with cognitive impairment is likely multifactorial. While there are key actions that hospital systems can take to create an age friendly perioperative care pathway, how well can our patients with cognitive impairment navigate that pathway? This study demonstrates that there is a clear difference in the ability to correctly execute preoperative instructions among those with impairment and suggests that this discrepancy likely persists with patient tasks throughout the perioperative spectrum. Notably, this was identified as modifiable—the key being concise tailored instruction and creating an active role for a patient-appointed perioperative partner. We hope this study prompts others to also assess their perioperative care pathway through the lens of the cognitively impaired patient.

## Data availability statement

The raw data supporting the conclusions of this article will be made available by the authors, without undue reservation.

## Ethics statement

The studies involving human participants were reviewed and approved by Institutional Review Board. The patients/participants provided their written informed consent to participate in this study.

## Author contributions

JD, AM, TG, and CK: study design and implementation. JD, PM, KT, and BW: data gathering. CM: data analysis. YM: primary author. JD: primary editor. All authors contributed to the article and approved the submitted version.

## Conflict of interest

The authors declare that the research was conducted in the absence of any commercial or financial relationships that could be construed as a potential conflict of interest.

## Publisher’s note

All claims expressed in this article are solely those of the authors and do not necessarily represent those of their affiliated organizations, or those of the publisher, the editors and the reviewers. Any product that may be evaluated in this article, or claim that may be made by its manufacturer, is not guaranteed or endorsed by the publisher.
